# CPAP Therapy Improves Intractable Hemifacial Spasm

**DOI:** 10.1155/2015/846234

**Published:** 2015-10-15

**Authors:** Narongrit Kasemsap, Sittichai Netwijitpan, Panita Limpawattana, Kannikar Kongbunkiat, Somsak Tiamkao, Verajit Chotmongkol, Noppadol Aekphachaisawat, Kittisak Sawanyawisuth

**Affiliations:** ^1^Department of Medicine, Faculty of Medicine, Khon Kaen University, Khon Kaen 40002, Thailand; ^2^North-Eastern Stroke Research Group, Khon Kaen University, Khon Kaen 40002, Thailand; ^3^Central Library, Silpakorn University, Bangkok 10400, Thailand; ^4^The Research and Training Center for Enhancing Quality of Life of Working-Age People, Khon Kaen University, Khon Kaen 40002, Thailand

## Abstract

The correlation between obstructive sleep apnea (OSA) and hemifacial spasm has never been reported in the literature. Here, we report a case of OSA-induced hypertension with intractable hemifacial spasm in which both conditions improved after continuous positive airway pressure treatment.

## 1. Introduction

Hemifacial spasm is a rare neuromuscular disease that causes abnormal spasm of facial muscles, particularly orbicularis oculi. The diagnosis can be made clinically through complete neurological examinations. Involuntary muscle contractions of the eyes lower patients' self-esteem and quality of life. Causes of the disease are still debated. Effective treatments include botulinum toxin injections and microvascular decompression of the facial nerve [1].

Obstructive sleep apnea (OSA) is a common sleep disorder. If untreated, OSA can cause several cardiovascular complications such as hypertension, stroke, or cardiac arrhythmia [2]. Continuous positive airway pressure (CPAP) machines have been shown to be effective in OSA treatment. Cardiovascular risks are significantly lowered with CPAP therapy. CPAP therapy can also convert atrial fibrillation to sinus rhythm in OSA-induced atrial fibrillation [3]. The correlation of OSA and hemifacial spasm has never been reported in the literature. Here, we report a case of intractable hemifacial spasm which was successfully treated with CPAP therapy.

## 2. A Case History

A 51-year-old Thai man was admitted to a hypertension/sleep clinic for hypertension workups. He noticed that his blood pressure had been high for months. He denied daytime sleepiness or other symptoms of OSA but noticed snoring and apneic events during the night. On physical examination, his blood pressure was 140/90 mmHg with a body mass index of 24 kg/m^2^. Oropharyngeal examination revealed macroglossia, mild retrognathia, Mallampati class 3, a neck circumference of 34 cm, and no tonsillar enlargement. He had no evidence of other secondary causes of hypertension. He also complained of intractable hemifacial spasm on his right side for seven years. His previous treatments included botulinum toxin and various medications such as muscle relaxants or anticonvulsants. Six years ago, magnetic resonance imaging of his brain showed that his anterior inferior cerebellar artery was attached to right exit zone of the facial nerve. Microvascular decompression surgery was performed six years ago but the symptom remained.

Laboratory workups for other causes of secondary hypertension were normal. Polysomnography was performed by Alice PDX due to a history of snoring and hypertension. His polysomnogram showed an apnea-hypopnea index (AHI) of 37.3 times/hour. In the total recording time of 479 minutes, an average oxygen saturation of 96% was found. Time with oxygen saturation lower than 95% was 75.2 minutes or 15.7%, and his lowest oxygen saturation was 83%. He was diagnosed as OSA-induced hypertension. CPAP therapy was introduced and was successful. He was able to use CPAP machine throughout the night with 90% of CPAP pressure of 7 cm H_2_O and the average AHI was less than 5 times/hour. At the successive one-month follow-ups, he reported that his hemifacial spasm symptoms had disappeared after CPAP therapy. His blood pressure was also under control. He was later given losartan 100 mg due to the presence of 128 mg of albuminuria per day. He is still compliant with CPAP therapy, and both hemifacial spasm and hypertension have remained under control after one year.

## 3. Discussion

It is unknown as to the reason that CPAP therapy improves intractable hemifacial spasm. There are several possible causes of hemifacial spasm, mostly due to facial nerve injuries or compression [1]. The main abnormality caused by OSA is intermittent hypoxemia. Hypoxemia may cause endothelial dysfunction or tissue hypoxemia [4]. We, therefore, believe that OSA may be a contributing factor to facial nerve ischemia, in particular in those who have some degree of facial nerve compression or injuries as did this patient.

Hemifacial spasm and OSA may be linked via hypertension. A case control study from Singapore showed that ventrolateral medulla (VLM) compression is associated with hypertension in hemifacial spasm [5]. Hypertensive hemifacial spasm patients and hypertensive controls significantly had VLM compression scores higher than healthy subjects. Microvascular decompression is effective for hemifacial spasm and patients who undergo the procedure rarely have recurrent hemifacial spasm symptoms. However, recurrent hemifacial spasm may occur in hypertensive patients as in this case [6]. As mentioned earlier, OSA is the most common cause of hypertension. Treating OSA with CPAP, therefore, improved hemifacial spasm, hypertension, and the OSA itself.

Further studies should be performed to explore the prevalence of OSA in hemifacial spasm patients and also the effectiveness of CPAP therapy in hemifacial spasm patients who have OSA as a comorbid disease. CPAP therapy may be a noninvasive treatment option for intractable hemifacial spasm patients. In addition, recurrent or intractable hemifacial spasm may have OSA as a comorbid disease particularly if hypertension is present.
